# (*E*)-2-(3-Chloro­benzyl­idene)-5,6-dimeth­oxy-2,3-dihydro-1*H*-inden-1-one

**DOI:** 10.1107/S1600536810040869

**Published:** 2010-10-20

**Authors:** Mohamed Ashraf Ali, Rusli Ismail, Soo Choon Tan, Chin Sing Yeap, Hoong-Kun Fun

**Affiliations:** a Institute for Research in Molecular Medicine, Universiti Sains Malaysia, 11800 USM, Penang, Malaysia; b X-ray Crystallography Unit, School of Physics, Universiti Sains Malaysia, 11800 USM, Penang, Malaysia

## Abstract

In the title compound, C_18_H_15_ClO_3_, the dihydro­indenone group makes a dihedral angle of 8.56 (6)° with the bezene ring. In the crystal, the mol­ecules are inter­connected into a three-dimensional network *via* inter­molecular C—H⋯O hydrogen bonds. Weak C—H⋯π and π⋯π [centroid–centroid distances 3.6598 (9)–3.6913 (9) Å] inter­actions are also observed.

## Related literature

For general background to and the biological activity of chalcone derivatives, see: Marzinzik & Felder (1998 ▶); Srikanth & Castle (2005 ▶); Furusawa *et al.* (2005 ▶) Heidenreich *et al.* (2008 ▶); Syed *et al.* (2008 ▶). For related structures, see: Ali *et al.* (2010*a* ▶,*b* ▶). For the stability of the temperature controller used in the data collection, see: Cosier & Glazer (1986 ▶).
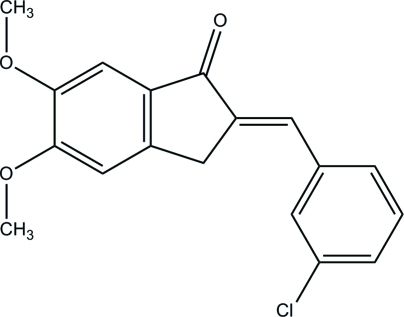


## Experimental

### 

#### Crystal data

C_18_H_15_ClO_3_
*M* *_r_* = 314.75
Tetragonal, 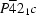

*a* = 20.5004 (16) Å
*c* = 7.0241 (7) Å
*V* = 2952.0 (4) Å^3^
*Z* = 8
Mo *K*α radiation
μ = 0.27 mm^−1^
*T* = 100 K
0.74 × 0.13 × 0.11 mm

#### Data collection

Bruker APEXII DUO CCD area-detector diffractometer
Absorption correction: multi-scan (*SADABS*; Bruker, 2009 ▶) *T*_min_ = 0.826, *T*_max_ = 0.971
62722 measured reflections
4499 independent reflections
4251 reflections with *I* > 2σ(*I*)
*R*_int_ = 0.045

#### Refinement

*R*[*F* ^2^ > 2σ(*F* ^2^)] = 0.031
*wR*(*F* ^2^) = 0.084
*S* = 1.06
4499 reflections
259 parameters
H atoms treated by a mixture of independent and constrained refinement
Δρ_max_ = 0.37 e Å^−3^
Δρ_min_ = −0.19 e Å^−3^
Absolute structure: Flack (1983 ▶), 1966 Friedel pairs
Flack parameter: −0.01 (5)

### 

Data collection: *APEX2* (Bruker, 2009 ▶); cell refinement: *SAINT* (Bruker, 2009 ▶); data reduction: *SAINT*; program(s) used to solve structure: *SHELXTL* (Sheldrick, 2008 ▶); program(s) used to refine structure: *SHELXTL*; molecular graphics: *SHELXTL*; software used to prepare material for publication: *SHELXTL* and *PLATON* (Spek, 2009 ▶).

## Supplementary Material

Crystal structure: contains datablocks global, I. DOI: 10.1107/S1600536810040869/sj5044sup1.cif

Structure factors: contains datablocks I. DOI: 10.1107/S1600536810040869/sj5044Isup2.hkl

Additional supplementary materials:  crystallographic information; 3D view; checkCIF report

## Figures and Tables

**Table 1:** Hydrogen-bond geometry (Å, °) *Cg*2 is the centroid of the C1–C6 ring.

*D*—H⋯*A*	*D*—H	H⋯*A*	*D*⋯*A*	*D*—H⋯*A*
C7—H7⋯O1^i^	0.942 (16)	2.489 (18)	3.2650 (16)	139.6 (15)
C11—H11⋯O1^ii^	0.951 (18)	2.561 (17)	3.3229 (16)	137.3 (14)
C18—H18*C*⋯O3^iii^	0.96 (2)	2.53 (2)	3.4684 (17)	165.2 (17)
C3—H3⋯*Cg*2^iv^	0.87 (2)	2.86 (2)	3.6072 (17)	144.4 (17)

Symmetry codes: (i) 
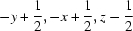
; (ii) 
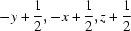
; (iii) 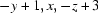; (iv) 
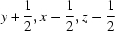
.

**Fig. 1.**
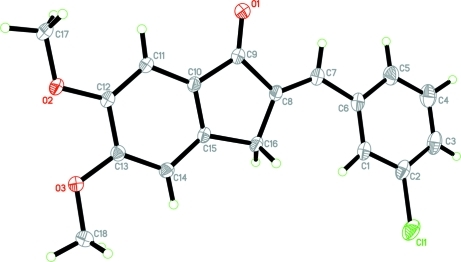
The molecular structure of the title compound with atom labels and 50% probability ellipsoids for non-H atoms.

**Fig. 2.**
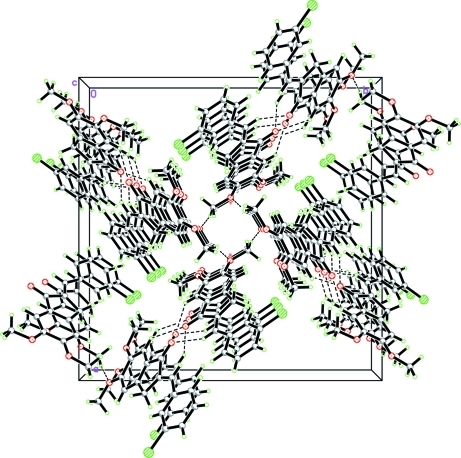
The crystal packing of title compound, showing a three-dimensional network. Intermolecular hydrogen bonds are shown as dashed lines.

## supplementary crystallographic information

### Comment

α,β-unsaturated ketones are useful key intermediates (Marzinzik & Felder, 1998, Srikanth & Castle, 2005) bearing the well known chalcone pharmacophore. Chalcones can be isolated from several plants and are precursors of flavones and anthocyan compounds. Some of them also exhibit antioxidant and anticancer properties. In fact, the pharmacological properties of chalcones are due to the presence of both α,β-unsaturation (Furusawa *et al.*, 2005) and an aromatic ring. Many antitumor drugs have been developed for prostate cancer patients, but their intolerable systemic toxicity often limits their clinical use. Chemoprevention is one of the most promising approaches in prostate cancer research, in which natural or synthetic agents are used to prevent this malignant disease (Heidenreich *et al.*, 2008, Syed *et al.*, 2008).

The molecular structure of the title compound is essentially planar (Fig. 1). The torsion angles of the two methoxy groups are [C18–O3–C13–C14] 4.38 (18) and [C17–O2–C12–C11] -2.01 (17)°. The maximum deviation of the dihydroindenone group is 0.024 (1) Å and it makes dihedral angle of 8.56 (6)° with the benzene ring [C1–C6]. The geometric parameters are comparable to those observed in closely related structures (Ali *et al.*, 2010*a*,*b*).

In the crystal structure, the molecules are linked together into a three dimensional network by the intermolecular C7—H7···O1, C11—H11···O1 and C18—H18C···O3 hydrogen bonds (Fig. 2, Table 1). Weak C—H···π and π···π interactions are also observed [*Cg*1···*Cg*2^v^ of 3.6913 (9) Å and *Cg*2···*Cg*3^vi^ of 3.6598 (9) Å; (v) *x*, *y*, 1 + *z*; (vi) *x*, *y*, -1 + *z. Cg*1, *Cg*2 and *Cg*3 are centroids of C8–C10/C15–C16, C1–C6 and C10–C15 rings, respectively].

### Experimental

A mixture of 5,6-dimethoxy-2,3-dihydro-1*H*-indene-1-one (0.001 mmol) and 3-chlorobenzaldehyde (0.001 mmol) were dissolved in methanol (10 ml) and 30% sodium hydroxide solution (5 ml) was added and the mixture stirred for 5 h. After the completion of the reaction as evident from TLC, the mixture was poured into crushed ice then neutralized with concentrated HCl. The precipitated solid was filtered, washed with water and recrystallized from ethanol to reveal the title compound as light yellow crystals.

### Refinement

All hydrogen atoms were located from difference Fourier map and refined freely. A total of 1966 Friedel pairs were use to determine the absolute structure.

### Crystal data

**Table:**

C_18_H_15_ClO_3_	*D*_x_ = 1.416 Mg m^−^^3^
*M**_r_* = 314.75	Mo *K*α radiation, λ = 0.71073 Å
Tetragonal, *P*42_1_*c*	Cell parameters from 9872 reflections
Hall symbol: P -4 2n	θ = 2.8–30.4°
*a* = 20.5004 (16) Å	µ = 0.27 mm^−^^1^
*c* = 7.0241 (7) Å	*T* = 100 K
*V* = 2952.0 (4) Å^3^	Needle, yellow
*Z* = 8	0.74 × 0.13 × 0.11 mm
*F*(000) = 1312	

### Data collection

**Table:**

Bruker APEXII DUO CCD area-detector diffractometer	4499 independent reflections
Radiation source: fine-focus sealed tube	4251 reflections with *I* > 2σ(*I*)
graphite	*R*_int_ = 0.045
φ and ω scans	θ_max_ = 30.5°, θ_min_ = 2.0°
Absorption correction: multi-scan (*SADABS*; Bruker, 2009)	*h* = −29→29
*T*_min_ = 0.826, *T*_max_ = 0.971	*k* = −28→29
62722 measured reflections	*l* = −10→10

### Refinement

**Table:**

Refinement on *F*^2^	Secondary atom site location: difference Fourier map
Least-squares matrix: full	Hydrogen site location: inferred from neighbouring sites
*R*[*F*^2^ > 2σ(*F*^2^)] = 0.031	H atoms treated by a mixture of independent and constrained refinement
*wR*(*F*^2^) = 0.084	*w* = 1/[σ^2^(*F*_o_^2^) + (0.0483*P*)^2^ + 0.5709*P*] where *P* = (*F*_o_^2^ + 2*F*_c_^2^)/3
*S* = 1.06	(Δ/σ)_max_ = 0.001
4499 reflections	Δρ_max_ = 0.37 e Å^−^^3^
259 parameters	Δρ_min_ = −0.19 e Å^−^^3^
0 restraints	Absolute structure: Flack (1983), 1966 Friedel pairs
Primary atom site location: structure-invariant direct methods	Flack parameter: −0.01 (5)

### Special details

**Table:**

Experimental. The crystal was placed in the cold stream of an Oxford Cryosystems Cobra open-flow nitrogen cryostat (Cosier & Glazer, 1986) operating at 100.0 (1) K.
Geometry. All e.s.d.'s (except the e.s.d. in the dihedral angle between two l.s. planes) are estimated using the full covariance matrix. The cell e.s.d.'s are taken into account individually in the estimation of e.s.d.'s in distances, angles and torsion angles; correlations between e.s.d.'s in cell parameters are only used when they are defined by crystal symmetry. An approximate (isotropic) treatment of cell e.s.d.'s is used for estimating e.s.d.'s involving l.s. planes.
Refinement. Refinement of *F*^2^ against ALL reflections. The weighted *R*-factor *wR* and goodness of fit *S* are based on *F*^2^, conventional *R*-factors *R* are based on *F*, with *F* set to zero for negative *F*^2^. The threshold expression of *F*^2^ > σ(*F*^2^) is used only for calculating *R*-factors(gt) *etc*. and is not relevant to the choice of reflections for refinement. *R*-factors based on *F*^2^ are statistically about twice as large as those based on *F*, and *R*- factors based on ALL data will be even larger.

### Fractional atomic coordinates and isotropic or equivalent isotropic displacement parameters (Å^2^)

**Table:**

	*x*	*y*	*z*	*U*_iso_*/*U*_eq_	
Cl1	0.658402 (17)	0.24083 (2)	0.07331 (5)	0.03382 (9)	
O1	0.35460 (5)	0.18458 (5)	0.76502 (14)	0.0273 (2)	
O2	0.38949 (4)	0.34023 (5)	1.38581 (13)	0.02125 (18)	
O3	0.49925 (5)	0.39341 (5)	1.30773 (13)	0.02196 (19)	
C1	0.55261 (6)	0.20587 (6)	0.27855 (18)	0.0204 (2)	
C2	0.59058 (6)	0.19228 (7)	0.11923 (18)	0.0235 (3)	
C3	0.57575 (8)	0.14194 (8)	−0.0056 (2)	0.0300 (3)	
C4	0.52078 (9)	0.10399 (8)	0.0310 (2)	0.0326 (3)	
C5	0.48177 (8)	0.11719 (7)	0.1883 (2)	0.0275 (3)	
C6	0.49729 (7)	0.16787 (6)	0.31451 (17)	0.0206 (2)	
C7	0.45338 (6)	0.17840 (6)	0.47637 (17)	0.0207 (2)	
C8	0.45819 (6)	0.21831 (6)	0.62791 (17)	0.0183 (2)	
C9	0.40455 (6)	0.21715 (6)	0.77302 (17)	0.0190 (2)	
C10	0.42366 (6)	0.26270 (6)	0.92367 (16)	0.0166 (2)	
C11	0.38957 (6)	0.27681 (6)	1.09221 (17)	0.0173 (2)	
C12	0.41636 (6)	0.32139 (6)	1.21623 (17)	0.0169 (2)	
C13	0.47750 (6)	0.35135 (6)	1.17248 (16)	0.0168 (2)	
C14	0.51055 (6)	0.33681 (6)	1.00419 (17)	0.0168 (2)	
C15	0.48283 (6)	0.29164 (6)	0.87923 (16)	0.0156 (2)	
C16	0.50915 (6)	0.26751 (6)	0.68977 (16)	0.0178 (2)	
C17	0.32745 (6)	0.31173 (7)	1.4304 (2)	0.0237 (2)	
C18	0.55815 (7)	0.42822 (7)	1.2670 (2)	0.0266 (3)	
H1	0.5664 (9)	0.2390 (9)	0.359 (3)	0.029 (5)*	
H3	0.6019 (10)	0.1348 (10)	−0.102 (3)	0.044 (6)*	
H4	0.5118 (11)	0.0700 (10)	−0.069 (4)	0.051 (6)*	
H5	0.4430 (11)	0.0892 (10)	0.215 (3)	0.051 (6)*	
H7	0.4154 (8)	0.1528 (8)	0.465 (3)	0.025 (4)*	
H11	0.3500 (9)	0.2539 (8)	1.115 (3)	0.026 (4)*	
H14	0.5503 (8)	0.3565 (9)	0.974 (3)	0.024 (4)*	
H18A	0.5551 (10)	0.4571 (9)	1.158 (3)	0.031 (5)*	
H18B	0.5953 (9)	0.3989 (9)	1.237 (3)	0.031 (5)*	
H18C	0.5683 (11)	0.4553 (10)	1.375 (3)	0.047 (6)*	
H16A	0.5126 (8)	0.3044 (8)	0.600 (3)	0.020 (4)*	
H16B	0.5517 (8)	0.2491 (8)	0.703 (2)	0.020 (4)*	
H17A	0.3144 (9)	0.3303 (9)	1.556 (3)	0.031 (5)*	
H17B	0.2950 (8)	0.3251 (8)	1.341 (2)	0.020 (4)*	
H17C	0.3292 (8)	0.2645 (8)	1.439 (3)	0.022 (4)*	

### Atomic displacement parameters (Å^2^)

**Table:**

	*U*^11^	*U*^22^	*U*^33^	*U*^12^	*U*^13^	*U*^23^
Cl1	0.02695 (16)	0.0484 (2)	0.02614 (16)	0.00242 (14)	0.00952 (13)	0.00399 (15)
O1	0.0264 (5)	0.0360 (5)	0.0195 (4)	−0.0121 (4)	0.0032 (4)	−0.0053 (4)
O2	0.0214 (4)	0.0246 (4)	0.0178 (4)	−0.0037 (3)	0.0070 (3)	−0.0051 (3)
O3	0.0234 (4)	0.0250 (4)	0.0175 (4)	−0.0077 (4)	0.0032 (3)	−0.0038 (3)
C1	0.0232 (6)	0.0233 (6)	0.0147 (5)	0.0064 (4)	0.0015 (4)	0.0019 (4)
C2	0.0227 (6)	0.0293 (6)	0.0184 (5)	0.0103 (5)	0.0031 (4)	0.0062 (5)
C3	0.0372 (8)	0.0338 (7)	0.0189 (5)	0.0146 (6)	0.0066 (6)	−0.0015 (5)
C4	0.0449 (8)	0.0292 (7)	0.0238 (6)	0.0079 (6)	0.0037 (6)	−0.0084 (5)
C5	0.0341 (7)	0.0271 (6)	0.0213 (6)	0.0015 (5)	0.0031 (5)	−0.0048 (5)
C6	0.0241 (6)	0.0228 (6)	0.0150 (5)	0.0053 (4)	0.0005 (4)	0.0008 (4)
C7	0.0234 (6)	0.0235 (6)	0.0154 (5)	0.0006 (5)	0.0024 (4)	−0.0003 (4)
C8	0.0197 (5)	0.0213 (5)	0.0139 (5)	−0.0001 (4)	0.0024 (4)	0.0001 (4)
C9	0.0205 (5)	0.0222 (5)	0.0144 (5)	−0.0021 (4)	0.0022 (4)	−0.0004 (4)
C10	0.0176 (5)	0.0191 (5)	0.0130 (4)	0.0002 (4)	0.0010 (4)	0.0005 (4)
C11	0.0173 (5)	0.0195 (5)	0.0152 (5)	−0.0011 (4)	0.0027 (4)	−0.0001 (4)
C12	0.0179 (5)	0.0182 (5)	0.0148 (5)	0.0008 (4)	0.0027 (4)	0.0006 (4)
C13	0.0184 (5)	0.0172 (5)	0.0150 (5)	0.0000 (4)	−0.0003 (4)	0.0011 (4)
C14	0.0155 (5)	0.0186 (5)	0.0163 (4)	0.0004 (4)	0.0003 (4)	0.0013 (4)
C15	0.0166 (5)	0.0180 (5)	0.0122 (4)	0.0018 (4)	0.0012 (4)	0.0015 (4)
C16	0.0183 (5)	0.0216 (5)	0.0136 (5)	0.0005 (4)	0.0037 (4)	0.0004 (4)
C17	0.0206 (6)	0.0310 (6)	0.0195 (5)	−0.0026 (5)	0.0064 (5)	−0.0010 (5)
C18	0.0240 (6)	0.0327 (7)	0.0232 (6)	−0.0098 (5)	0.0013 (5)	−0.0047 (5)

### Geometric parameters (Å, °)

**Table:**

Cl1—C2	1.7401 (15)	C8—C16	1.5160 (17)
O1—C9	1.2237 (15)	C9—C10	1.4647 (16)
O2—C12	1.3680 (14)	C10—C15	1.3860 (16)
O2—C17	1.4343 (15)	C10—C11	1.4048 (16)
O3—C13	1.3582 (14)	C11—C12	1.3768 (17)
O3—C18	1.4314 (16)	C11—H11	0.951 (18)
C1—C2	1.3914 (17)	C12—C13	1.4293 (16)
C1—C6	1.3988 (19)	C13—C14	1.3948 (16)
C1—H1	0.926 (19)	C14—C15	1.3968 (16)
C2—C3	1.388 (2)	C14—H14	0.935 (17)
C3—C4	1.393 (2)	C15—C16	1.5188 (16)
C3—H3	0.87 (2)	C16—H16A	0.989 (17)
C4—C5	1.390 (2)	C16—H16B	0.955 (17)
C4—H4	1.01 (2)	C17—H17A	0.994 (19)
C5—C6	1.4025 (18)	C17—H17B	0.953 (17)
C5—H5	1.00 (2)	C17—H17C	0.971 (17)
C6—C7	1.4661 (17)	C18—H18A	0.97 (2)
C7—C8	1.3461 (17)	C18—H18B	0.994 (19)
C7—H7	0.943 (17)	C18—H18C	0.96 (2)
C8—C9	1.4995 (16)		
			
C12—O2—C17	115.60 (10)	C12—C11—H11	124.0 (11)
C13—O3—C18	116.98 (10)	C10—C11—H11	117.8 (11)
C2—C1—C6	119.16 (12)	O2—C12—C11	125.30 (11)
C2—C1—H1	117.7 (12)	O2—C12—C13	114.76 (10)
C6—C1—H1	123.1 (12)	C11—C12—C13	119.94 (11)
C3—C2—C1	122.31 (14)	O3—C13—C14	124.68 (11)
C3—C2—Cl1	118.89 (11)	O3—C13—C12	114.23 (10)
C1—C2—Cl1	118.79 (11)	C14—C13—C12	121.09 (11)
C2—C3—C4	118.40 (13)	C13—C14—C15	118.47 (11)
C2—C3—H3	118.6 (14)	C13—C14—H14	121.7 (12)
C4—C3—H3	123.0 (14)	C15—C14—H14	119.9 (12)
C5—C4—C3	120.22 (14)	C10—C15—C14	119.89 (11)
C5—C4—H4	125.7 (14)	C10—C15—C16	111.65 (10)
C3—C4—H4	113.9 (14)	C14—C15—C16	128.46 (11)
C4—C5—C6	121.06 (14)	C8—C16—C15	102.88 (9)
C4—C5—H5	119.9 (13)	C8—C16—H16A	112.0 (10)
C6—C5—H5	119.0 (13)	C15—C16—H16A	109.8 (10)
C1—C6—C5	118.84 (12)	C8—C16—H16B	113.2 (10)
C1—C6—C7	123.76 (12)	C15—C16—H16B	111.7 (10)
C5—C6—C7	117.39 (12)	H16A—C16—H16B	107.4 (14)
C8—C7—C6	131.13 (12)	O2—C17—H17A	106.0 (11)
C8—C7—H7	117.8 (11)	O2—C17—H17B	111.0 (10)
C6—C7—H7	111.1 (11)	H17A—C17—H17B	106.4 (14)
C7—C8—C9	118.31 (11)	O2—C17—H17C	112.8 (10)
C7—C8—C16	132.96 (11)	H17A—C17—H17C	109.7 (15)
C9—C8—C16	108.73 (10)	H17B—C17—H17C	110.5 (14)
O1—C9—C10	127.22 (11)	O3—C18—H18A	114.1 (12)
O1—C9—C8	126.23 (11)	O3—C18—H18B	112.8 (11)
C10—C9—C8	106.55 (10)	H18A—C18—H18B	104.5 (15)
C15—C10—C11	122.49 (11)	O3—C18—H18C	108.2 (14)
C15—C10—C9	110.14 (10)	H18A—C18—H18C	106.5 (16)
C11—C10—C9	127.37 (11)	H18B—C18—H18C	110.5 (17)
C12—C11—C10	118.13 (11)		
			
C6—C1—C2—C3	−0.32 (19)	C9—C10—C11—C12	179.81 (12)
C6—C1—C2—Cl1	178.78 (10)	C17—O2—C12—C11	−2.01 (17)
C1—C2—C3—C4	0.0 (2)	C17—O2—C12—C13	178.74 (11)
Cl1—C2—C3—C4	−179.06 (12)	C10—C11—C12—O2	−179.80 (11)
C2—C3—C4—C5	0.7 (2)	C10—C11—C12—C13	−0.59 (17)
C3—C4—C5—C6	−1.1 (2)	C18—O3—C13—C14	4.38 (18)
C2—C1—C6—C5	−0.09 (19)	C18—O3—C13—C12	−176.10 (11)
C2—C1—C6—C7	−179.15 (12)	O2—C12—C13—O3	0.50 (15)
C4—C5—C6—C1	0.8 (2)	C11—C12—C13—O3	−178.79 (11)
C4—C5—C6—C7	179.91 (14)	O2—C12—C13—C14	−179.96 (11)
C1—C6—C7—C8	−7.7 (2)	C11—C12—C13—C14	0.74 (18)
C5—C6—C7—C8	173.26 (14)	O3—C13—C14—C15	178.91 (11)
C6—C7—C8—C9	−179.20 (12)	C12—C13—C14—C15	−0.58 (17)
C6—C7—C8—C16	0.6 (2)	C11—C10—C15—C14	−0.16 (18)
C7—C8—C9—O1	−2.7 (2)	C9—C10—C15—C14	−179.73 (11)
C16—C8—C9—O1	177.46 (13)	C11—C10—C15—C16	179.72 (10)
C7—C8—C9—C10	177.72 (11)	C9—C10—C15—C16	0.15 (14)
C16—C8—C9—C10	−2.16 (13)	C13—C14—C15—C10	0.29 (17)
O1—C9—C10—C15	−178.36 (13)	C13—C14—C15—C16	−179.57 (11)
C8—C9—C10—C15	1.25 (13)	C7—C8—C16—C15	−177.69 (13)
O1—C9—C10—C11	2.1 (2)	C9—C8—C16—C15	2.15 (12)
C8—C9—C10—C11	−178.30 (11)	C10—C15—C16—C8	−1.44 (13)
C15—C10—C11—C12	0.32 (18)	C14—C15—C16—C8	178.43 (12)

### Hydrogen-bond geometry (Å, °)

**Table:**

*Cg*2 is the centroid of the C1–C6 ring.

**Table:**

*D*—H···*A*	*D*—H	H···*A*	*D*···*A*	*D*—H···*A*
C7—H7···O1^i^	0.942 (16)	2.489 (18)	3.2650 (16)	139.6 (15)
C11—H11···O1^ii^	0.951 (18)	2.561 (17)	3.3229 (16)	137.3 (14)
C18—H18C···O3^iii^	0.96 (2)	2.53 (2)	3.4684 (17)	165.2 (17)
C3—H3···Cg2^iv^	0.87 (2)	2.86 (2)	3.6072 (17)	144.4 (17)

Symmetry codes: (i) −*y*+1/2, −*x*+1/2, *z*−1/2; (ii) −*y*+1/2, −*x*+1/2, *z*+1/2; (iii) −*y*+1, *x*, −*z*+3; (iv) *y*+1/2, *x*−1/2, *z*−1/2.
